# Fluoroquinolones and Aortic Diseases: Is There a Connection

**DOI:** 10.1055/s-0039-1693468

**Published:** 2019-09-17

**Authors:** Davide Carino, Mohammad A. Zafar, Mrinal Singh, Bulat A. Ziganshin, John A. Elefteriades

**Affiliations:** 1 Department of Surgery, Aortic Institute at Yale-New Haven, Yale University School of Medicine, New Haven, Connecticut

**Keywords:** aortic disease, aortic dissection, fluoroquinolones

## Abstract

Fluoroquinolones (FQs) are one of the most commonly prescribed classes of antibiotics. Their high tissue distribution and broad-spectrum antibacterial coverage make their use very attractive in numerous infectious diseases. Although generally well tolerated, FQs have been associated with different adverse effects including dysglycemia and arrhythmias. FQs have been also associated with a series of adverse effects related to collagen degradation, such as Achilles tendon rupture and retinal detachment. Recently, an association between consumption of FQs and increased risk of aortic aneurysm and dissection has been proposed. This article reviews the pathogenesis of thoracic aortic diseases, the molecular mechanism of FQ-associated collagen toxicity, and the possible contribution of FQs to aortic diseases.

## Introduction

Fluoroquinolones (FQs) were introduced in the 1980s and since then have been used extensively to treat a wide range of infections. FQs directly inhibit bacterial deoxyribonucleic acid (DNA) synthesis by binding two bacterial enzymes: DNA gyrase and topoisomerase IV. 1 FQs possess favorable pharmacokinetics properties, particularly good tissue penetration that is reflected by their high volume of distribution. 1 First-generation FQs are especially active against Gram-negative organisms, while the “new generation” FQs possess improved activity against Gram-positive and anerobic bacteria. 2 Their favorable pharmacokinetics characteristics and broad-spectrum activity make this class of antibiotics one of the most used in North America. 3 4 The United States FQ usage statistics are staggering:

- FQs have become the most commonly prescribed antibiotic class 3
- A total of 22 million FQ prescriptions are written per year 3 4
- About 24% of all antibiotic prescriptions written are for FQs 3 4
- FQs have been administered to 10% of adults. 3 4

FQs include the narrower-spectrum drugs such as ciprofloxacin, ofloxacin, norfloxacin, and lomefloxacin and the broader-spectrum drugs such as levofloxacin, trovafloxacin, gatifloxacin, grepafloxacin, and moxifloxacin.

Overall, FQs are well tolerated. The most common adverse effects involve the gastrointestinal tract (nausea and diarrhea) and the central nervous system (headache and dizziness). 5 These side effects are usually extremely mild and do not necessitate interruption of therapy. Some FQs are associated with prolongation of the QT interval, 6 in particular moxifloxacin. This drug should be used with caution in patients with predisposing factors for *torsades de pointes* .

One of the most worrisome adverse effects of FQs is atraumatic rupture of the Achilles tendon, first reported in 1983. 7 Since then, the number of clinical reports concerning tendinopathies associated with FQs therapy has been increasing, reflecting the wider use of these drugs as well as greater awareness of this unusual adverse reaction. Large epidemiological studies have confirmed this association, 8 9 with most of the cases reported in patients older than 60 years of age. 10

The deleterious effect of FQs on collagen is not limited to tendons. Collagen plays a critical role in maintaining retinal attachment and is integral to the structure of the vitreous body. 11 Thus, it has been hypothesized that the degradation of collagen associated with the consumption of FQs may promote the development of posterior vitreous detachment, leading to an increased risk of retinal detachment. This was demonstrated in population studies in Canada and Taiwan, 12 with an adjusted rate ratio (RR) of 4.50. Other studies, however, have failed to show a relationship. 13 This possible association is still in the investigational phase.

Collagen is abundant in the aortic wall, and the substantiated deleterious effects of FQs on collagen raise the concern for a contributory role in aortic aneurysm or dissection. An association between the progression of thoracic aortic aneurysm (TAA) and FQ consumption has recently been proposed 9 as well as an increased risk of acute aortic dissection (AAD). 14 15 16

In this report, we review the pathogenesis of TAA and AAD, analyze the molecular mechanisms that may explain FQ-associated collagen toxicity, and explore how FQs may be linked to thoracic aortic disease.

## Ultrastructural Anatomy of Tendon and Aortic Wall

Tendons are largely composed of collagen, accounting for 70% of their dry weight. 17 Almost 90% of this collagen is Type I and the remaining 10% is Type III. 18 Type I collagen is organized into fibrils. Fibrils are grouped in parallel to form bundles. Type III collagen is found in the endotendineum that surrounds the bundles. Collagen is produced by fibroblasts, while its physiologic degradation is determined by the balance between the activity of two groups of enzymes: matrix metalloproteinases (MMPs) and tissue inhibitors of the MMPs (TIMPs). 19 The MMPs are a group of zinc-dependent enzymes with collagenolytic activity. There exist many different types of MMPs, each with a specific tissue distribution. In tendons, the most active are MMP-2 and MMP-9, 19 with activity on both Type I and Type III collagen. Furthermore, MMPs are also able to degrade elastin. 20 TIMPs are a family of enzymes capable of inhibiting the activity of the MMPs. 21 There are four members of the TIMP family.

Collagen is also a major component of the aortic wall. The aortic wall is divided into three layers: intima, media, and adventitia. The endothelium is the innermost layer of the intima and forms a barrier between the structural aorta and the passing blood. Only a small amount of collagen is present in the intima, mainly Type III. 22 The intima is sharply demarcated from the media by the internal elastic lamina. The media is the thickest of the layers of the aortic wall, composed of circumferentially arranged lamellar units. Lamellar units consist of a layer of elastic fibers overlying a layer of smooth muscle cells. 23 These lamellae can be considered to be the functional units of the media. Within the lamellar units lies extracellular matrix material, composed predominantly of collagen. Mucopolysaccharides and glycosaminoglycans are present as well. 24 The collagen is mainly Type III, but Types I and IV are also present. 22 The integrity of the network of the lamellar units is responsible for the physical characteristics of the aorta, 25 permitting the physiological changes that occur during the cardiac cycle. During systole, the aortic diameter increases and the wall thickness decreases, and vice versa during diastole. 26

TAAs are characterized by qualitative and quantitative defects in lamellar units. 25 A sharp deterioration of the normal mechanical properties of the aorta in patients with TAA reflects the structural deficits. 26 These alterations produce an increase in wall stress, which underlies, in engineering terms, the malignant clinical behavior (rupture or dissection) of TAAs.

## Pathogenesis of Thoracic Aortic Disease

Progress over the last 20 years has added much to our understanding of natural history, genetic basis, and molecular pathogenesis of thoracic aorta diseases. 27 This work can be summarized by answering the following questions:

1. *Genetics* : Is TAA a genetic disease?
2. *Molecular pathogenesis* : What is the molecular pathogenesis of TAA?
3. *Inciting events* : How does AAD pick one point in time at which to occur?

1. *Genetics* : Through analysis of family trees of the patients in our database, our group noticed that more than 20% of the patients operated for TAA or AAD had at least one family member with aortic aneurysm. 28 In addition to a family pattern of inheritance, we also noticed that patients with positive family history manifest higher rate of growth of the aorta and present at an earlier age. 29 Moreover, the true incidence of parent to child transmission is likely greater than 20% since many family members may not be aware of their aneurysm. 27 With growing patient number and duration of follow-up, our database, which now contains more than 4,000 patients and 12,000 patient-years of follow-up, consistently demonstrates that TAA is a genetic disease. 30 The pioneering work of Milewicz 31 and others has determined the specific genetic mutations underlying the familial pattern of transmission.
2. *Molecular pathogenesis* : In 2004, we demonstrated a local increase in MMP activity (specifically Type 2 and Type 9) together with an increased MMP-9 to TIMP-1 ratio, in aortic specimens of patients with TAA and AAD. 32 This was subsequently corroborated by the Houston group 33 and others. 34 Increased activity of MMPs in the aortic wall causes a destruction of collagen and elastin in the extracellular matrix of the media. This results in derangement of the network of the lamellar unit characteristic of the TAA. 25 Notably, patients with AAD had even significantly higher MMP expression than patients with (nondissected) TAA. 32
3. *Inciting events* : In 2000, we noticed the occurrence of AAD in five young athletes during high- intensity weightlifting or other strenuous exercises. 35 After the publication of our paper, we received other similar reports nationwide. 36 A subsequent study by our group demonstrated extremely marked rises in blood pressure during weightlifting in healthy volunteers. 30 Furthermore, we demonstrated that in more than ⅔ of a wide group of patients who suffered AAD, a severe physical or emotional stress (with an almost certain subsequent hypertensive reaction) preceded the onset of the dissection pain. 37 We therefore postulate that acute AAD is precipitated by a specific, severe hypertensive event that increases aortic wall stress beyond the tensile limit of the structurally impaired aorta, resulting in disruption of layers (i.e., AAD).

**Fig. 1:**
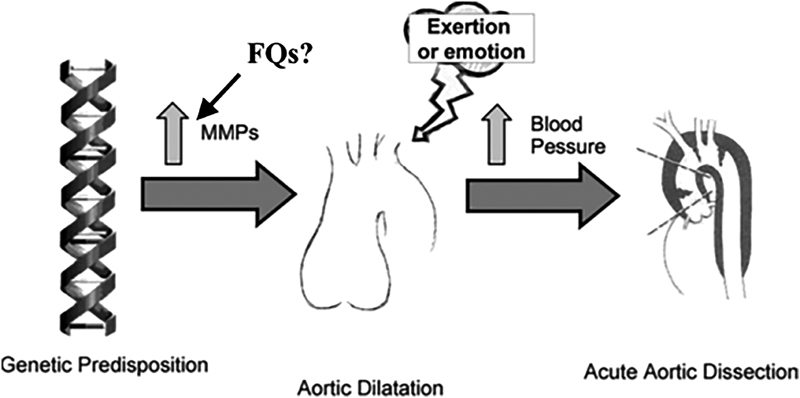
Schematic of pathophysiologic processes leading to acute aortic dissection at one specific point in time. (Reproduced with permission from Elefteriades. 30 ).FQ, fluoroquinolone; MMP, matrix metalloproteinases.

On the basis of our findings, we hypothesize the following schema for the genesis and timing of AAD ( Fig. 1 ):

- Susceptibility to TAA is genetically determined;
- Excess MMP activity in the vessel wall disrupts the integrity of the lamellar units, leading to progressive aortic dilatation;
- Ultrastructural changes combined with dilatation result in deterioration of the aorta's mechanical proprieties;
- A specific, hypertensive event associated with exertion or emotion increases aortic wall stress beyond its tensile limit, inducing AAD.

## Recent Population-Based Studies Relating Fluoroquinolones to Aortic Events

Different investigators have chosen different imaginative population-based methods to explore the impact of FQ treatment on various important clinical events.

Daneman (2015) 9 (Treatment versus nontreatment periods.): This Canadian national database study followed 657,950 older adults (>65 years) who received at least one FQ prescription until primary outcome (tendon rupture, retinal detachment, or aortic event), death, or loss to follow-up. Rates of events were 2.1% for tendon rupture, 0.2% for retinal detachment, and 1.1% for aortic aneurysm. These events were more common during and early after treatment intervals than at other periods. FQs were associated with an increased hazard risk during treatment periods of 3.13 for tendon rupture, 1.28 for retinal detachment, and 2.72 for aortic aneurysm. Among the “aneurysm” events, 53% triggered emergency hospital admission and 17% of cases were admitted specifically for “rupture or dissection.”

Lee (2015) 14 (Case-control analysis.): This Taiwanese study compared 1,477 patients who experienced aortic aneurysm or dissection to 147,000 controls. They found an increased RR of 2.43.

Pasternak (2018) 16 (Fluoroquinolone treatment compared with amoxicillin treatment.): This Swedish database study compared propensity score for aortic aneurysm or dissection among 360,088 fluroquinolone treatment episodes to the same number of amoxicillin treatment episodes. The hazard ratio was 1.66 and the increase in aortic events was 66%. The increased incidence was most pronounced within the first 10 days from start of FQ treatment.

Lee (2018) 15 (Treatment versus nontreatment periods *.* ): In this study from Taiwan, the odds ratio (OR) for an aortic event was 2.71 time higher for the 60 days after FQ prescription than for an equivalent non-FQ period. The OR was higher (2.83) for prolonged FQ exposure (>14 days) than for shorter exposure (2.41).

These studies, 9 14 15 16 employing a variety of methods and populations, all showed relatively powerful suggestions of a link between FQ use and aortic events. Each method of analysis has strengths and weakness, and while the overall thrust is strong, unequivocal clinical proof of a causative connection cannot reasonably be claimed from these population studies alone. Also, these studies combine aortic aneurysm and aortic dissection as endpoints. For aortic specialists, this combination is unsatisfying, as “aortic aneurysm” is not normally pictured as a specific acute event, but rather as an anatomic development over a long period of time. Aortic dissection, on the other hand, represents an archetypal acute clinical event.

## Molecular Mechanism of FQ-Associated Collagen Toxicity

Increased awareness of FQ-associated tendinopathies has led many to investigate the molecular mechanism underlying this unusual adverse effect. Williams et al showed an increased proteolytic activity in canine Achilles tendon fibroblasts cultured with ciprofloxacin. 38 The proteolytic activity of the FQs on collagen has also been observed after systemic administration. Degeneration of the extracellular matrix with decreased diameter of collagen fibers and increased distance between collagen fibers in tendons of rats treated with FQs was seen by Shakibaei et al. 39 A further study revealed a dose-dependent, quantitative reduction in Type I collagen in human tendons cultured with FQs. 40 This study also showed a linear correlation between both the concentration of FQs and the duration of the incubation with the degradation of collagen and the MMP activity. In 2011, Tsai et al elegantly demonstrated that FQs cause an upregulation of the MMPs, (particularly Type II) at the mRNA and protein level, but without increased gene expression. 19 Others have shown FQ-induced upregulation of MMP Type I. 41 Very recently, LeMaire et al have shown that administration of ciprofloxacin to an aneurysm-prone mouse model dramatically increased the incidence and severity of aortic dissection and produced aneurysm-related death. 42 Furthermore, ciprofloxacin severely disrupted nuclear and mitochondrial function in cultured smooth muscle cells.

Ultimately spontaneous tendon rupture after consumption of FQs can be attributed to an increased activity of the MMPs, in particular the MMP Type I and Type II, resulting in excessive degradation of Type I and Type III collagen in the tendon (and other tissues). From a clinical perspective, an important finding from these studies is a linear relationship between duration of therapy and MMP activity, with subsequent risk of tendon rupture. Clinicians should be aware of this pertinent finding when prescribing prolonged courses of FQs.

## FQs and Achilles Tendon Rupture 43

Since the original reports in 1983, FQs have been consistently and convincingly associated with Achilles tendon rupture. Hundreds of cases have been reported. Case control studies have demonstrated an OR for Achilles tendon rupture (compared with a control population) of 4.1 44 and an estimated incidence in FQ-treated patients of 3.2 cases per 1,000 patient-years. 45 Athletes and the elderly are more susceptible, as are those concurrently taking steroid medications and patients with renal failure. 46 47

Symptoms may range from local pain (usually located 2–6 cm proximal to the insertion of the tendon) to frank rupture of the Achilles tendon.

**Fig. 2:**
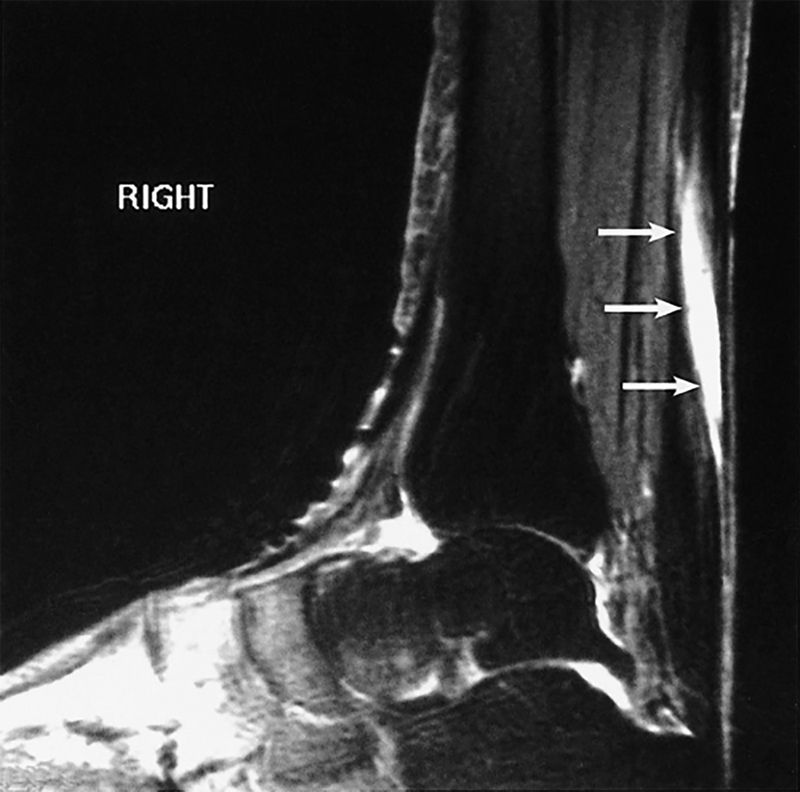
Magnetic resonance imaging of fluoroquinolone-induced Achilles tendon rupture. Sagittal T2-weighted fat-saturated image shows a high-grade tear at the musculotendinous junction. (Reproduced with permission from Yu C and Giuffre B. 48 )

Ultrasound and magnetic resonance imaging (MRI) can confirm the clinical diagnosis of Achilles tendonitis or Achilles tendon rupture. On MRI, areas of tendon degeneration are seen as zones of high signal intensity on T1- and T2-weighted images 48 as well as frank interruptions of tendon tissue ( Fig. 2 ).

The mean time of onset of Achilles tendon symptoms ranged from 2 hours to 6 months, with a mean onset 2 weeks after initiation of FQ therapy. 49

Other tendons have been affected, including the quadriceps and rotator cuff.

It is recommended that FQs be avoided in patients at risk for Achilles tendon rupture (including athletes and those on steroid medications). Similarly, it is recommended that once FQs have been administered, athletic schedules should be limited in duration and intensity (especially high-intensity and “ballistic” type activities).

Treatment ranges from rest (for less severe cases with predominantly inflammatory signs) to surgical repair (for severe rupture cases). At surgery, thickened, degenerated Achilles tendon tissues are found. It goes without saying that the offending FQ must be stopped, if it is still being administered at the time of clinical presentation with tendon impairment or rupture.

The association of FQ with these orthopaedic manifestations and tendon ruptures has been fully established and accepted by the medical community. 12 50

## What About Mitral Regurgitation?

Collagen is also an important component of mitral valve chordae tendineae. 51 Normal chordae tendineae have a central fibrous core-containing close-packed collagen fibrils. The central core is surrounded by a thin layer of elastic fibers and collagen fibrils, both of which are closely associated with proteoglycans. 51 The collagen is mainly Type III but Type I is present as well. 52 The ultrastructural integrity of the chordae imparts the tensile strength necessary for the repeated normal closure of the mitral valve, avoiding prolapse of the mitral leaflets. In myxomatous chordae, both the collagen and the elastin are deformed and fragmented. 52 These alterations cause elongation of the chordae and reduce the normal tensile strength, leading to mitral regurgitation secondary to leaflet prolapse or flail (due to chordal rupture). An imbalance between MMPs and TIMPs has been shown in animal models of myxomatous mitral valve regurgitation 53 54 as well as in surgically excised human myxomatous mitral valve leaflets. 55 56 57

To date, no data regarding any association between FQs and myxomatous mitral valve disease exists. We hypothesize that FQs could cause myxomatous mitral valve disease by inducing MMPs activity. Large retrospective epidemiological studies are required to investigate further.

## FQs and Thoracic Aortic Aneurysms and Acute Aortic Dissection

Given the importance of MMPs in TAA pathogenesis, it is possible that the increased activity of this group of enzymes (in particular MMP-2) induced by the FQs may promote the development of thoracic aortic disease. In particular, in susceptible patients, FQs could accelerate the derangement of the lamellar unit, leading to an acute deleterious change of the mechanical properties of the thoracic aorta, specifically lowering the tensile strength. A hypertensive event may then more easily trigger an AAD (as in Fig. 1 ).

Just as animal studies have shown a duration-dependent increase in MMP activity, so also human observations have shown an association between longer duration of FQ therapy and increased risk of AAD. 14 A recent nested case-control analysis showed that the current use (within 60 days) of a FQ increases the OR of aortic aneurysm or dissection to 2.43 and prior use (between 61 and 365 days) increases the OR by 1.48. 14

A multitude of legal sites have proliferated online and on television advertising for patients who have suffered aortic dissection and been treated with FQs. Whether a legal connection is supported will soon be determined in the courts.

## Conclusions

In conclusion, the ultrastructural similarity of tendon and aortic wall, together with FQ-induced MMP overactivity, may well explain the emerging association between these antibiotics and AAD 14 and TAA. 9 In 2016, the US Food and Drug Administration (FDA) enhanced its warning regarding the collagen-damaging effects of FQs, 58 although still falling short of implicating FQs in TAA and AAD. Most recently, on October 1, 2018, the European Medical Agency's Pharmacovigilance Risk Assessment Committee issued a recommendation to amend the product information characteristics of FQs for systemic and inhaled use with detailed information regarding the increased risk of aortic aneurysm and dissection. 59 Furthermore, on December 20, 2018, the FDA issued a formal warning regarding the increased risk of rupture or tears in the aorta with FQ antibiotics use in certain patients. 60

We recommend that cardiologists and cardiac surgeons increase their vigilance in looking for a connection of acute aortic events to FQs. We recommend extreme caution in the administration of FQ to patients with aortic disease. We look forward to further laboratory investigations and population studies on this important potential connection.
